# High susceptibility of metastatic cells derived from human prostate and colon cancer cells to TRAIL and sensitization of TRAIL-insensitive primary cells to TRAIL by 4,5-dimethoxy-2-nitrobenzaldehyde

**DOI:** 10.1186/1476-4598-10-46

**Published:** 2011-04-25

**Authors:** Hak-Bong Kim, Mi-Ju Kim, Dae-Young Kim, Jae-Won Lee, Jae-Ho Bae, Dong-Wan Kim, Chi-Dug Kang, Sun-Hee Kim

**Affiliations:** 1 Department of Biochemistry, Pusan National University School of Medicine, Yangsan 626-870, South Korea; 2 Research Center for Ischemic Tissue regeneration, Pusan National University School of Medicine, Yangsan 626-870, South Korea; 3 Department of Microbiology, College of Natural Sciences, Chang Won National University, Chang Won 641-773, South Korea

## Abstract

**Background:**

Tumor recurrence and metastasis develop as a result of tumors' acquisition of anti-apoptotic mechanisms and therefore, it is necessary to develop novel effective therapeutics against metastatic cancers. In this study, we showed the differential TRAIL responsiveness of human prostate adenocarcinoma PC3 and human colon carcinoma KM12 cells and their respective highly metastatic PC3-MM2 and KM12L4A sublines and investigated the mechanism underlying high susceptibility of human metastatic cancer cells to TRAIL.

**Results:**

PC3-MM2 and KM12L4A cells with high level of c-Myc and DNA-PKcs were more susceptible to TRAIL than their poorly metastatic primary PC3 and KM12 cells, which was associated with down-regulation of c-FLIP_L/S _and Mcl-1 and up-regulation of the TRAIL receptor DR5 but not DR4 in both metastatic cells. Moreover, high susceptibility of these metastatic cells to TRAIL was resulted from TRAIL-induced potent activation of caspase-8, -9, and -3 in comparison with their primary cells, which led to cleavage and down-regulation of DNA-PKcs. Knockdown of c-Myc gene in TRAIL-treated PC3-MM2 cells prevented the increase of DR5 cell surface expression, caspase activation and DNA-PKcs cleavage and attenuated the apoptotic effects of TRAIL. Moreover, the suppression of DNA-PKcs level with siRNA in the cells induced the up-regulation of DR5 and active caspase-8, -9, and -3. We also found that 4,5-dimethoxy-2-nitrobenzaldehyde (DMNB), a specific inhibitor of DNA-PK, potentiated TRAIL-induced cytotoxicity and apoptosis in relatively TRAIL-insensitive PC3 and KM12 cells and therefore functioned as a TRAIL sensitizer.

**Conclusion:**

This study showed the positive relationship between c-Myc expression in highly metastatic human prostate and colon cancer cells and susceptibility to TRAIL-induced apoptosis and therefore indicated that TRAIL might be used as an effective therapeutic modality for advanced metastatic cancers overexpressing c-Myc and combination of TRAIL therapy with agent that inhibits the DNA-PKcs/Akt signaling pathway might be clinically useful for the treatment of relatively TRAIL-insensitive human cancers.

## Background

Despite the improvement of therapeutic strategies against cancer, the acquisition of invasive/metastatic capabilities and the development of resistance to therapy in cancer cells are still critical problems for successful cancer therapy because recurrent or metastatic cancers that appear after the initial radiotherapy or chemotherapy are generally refractory to secondary therapies [1]. Some metastatic cancers are more resistant to chemotherapeutic drugs than their poorly metastatic counterparts as a result of their acquisition of anti-apoptotic mechanisms [2-5]. Therefore, it is necessary to elucidate the therapy-resistance mechanisms of metastatic cells for development of effective therapeutic modalities against metastatic cancers, since the molecular basis for the association of an aggressive metastatic phenotype with resistance to apoptosis is still unclear.

The death-inducing cytokine tumor necrosis factor (TNF)-related apoptosis-inducing ligand (TRAIL) holds enormous promise as an anti-cancer agent due to its highly selective apoptosis-inducing action on neoplastic versus normal cells [6,7]. The apoptosis signaling cascade is initiated through the engagement of the cell surface death receptors DR4 and/or DR5 by their ligand TRAIL. The binding of TRAIL to the death receptors leads to their trimerization and the recruitment of Fas-associated protein with death domain (FADD). Subsequently, FADD recruits the initiator procaspase-8 or -10, leading to the assembly of the death-inducing signaling complex (DISC), where the initiator caspases are autoactivated by proteolysis. Activated caspase-8 or -10 then cleaves the effector caspase-3, resulting in the cleavage of the death substrates [8].

c-Myc is deregulated and over-expressed in many cancer cells. The deregulation of c-Myc confers a selective advantage on cancer cells by promoting proliferation, cell survival, and genetic instability, which can contribute to metastasis [9]. By contrast, the activation of c-Myc dramatically sensitizes cells to the apoptotic action of TRAIL by up-regulating the cell surface level of DR5 and activating DISC, thereby playing an important role in determining cellular sensitivity to TRAIL [10]. The decision of a cell to undergo apoptosis or to promote cell survival by c-Myc depends on the specific cell type and the physiological status of the cell [11]. c-Myc-dependent priming of the mitochondrial pathway is critical for the capacity of TRAIL-induced caspase-8 signals to activate effector caspases and for the establishment of a lethal caspase feedback amplification loop in human cells [12,13].

Recently, we demonstrated that metastatic cancer cells have an increased level and activity of DNA-PKcs, the catalytic subunit of DNA-dependent protein kinase (DNA-PK), compared with their primary cells [14], and An et al. reported that DNA-PKcs regulates the stability of c-Myc through the Akt/GSK3β/c-Myc ubiquitination signal pathway [15]. In addition, it has been shown that the level of phosphorylated Ser-473 Akt was much higher in metastatic cancer cells than in non-metastatic cancer cells, and siRNA against Akt blocked cell migration, indicating that Akt activation is necessary for the metastasis of these cultured cells [16].

Therefore, we studied sensitivity of metastatic cancer cells to TRAIL and demonstrated the differential TRAIL responsiveness of two pairs of primary (PC3 and KM12) and metastatic (PC3-MM2 and KM12L4A) cells. PC3-MM2 and KM12L4A cells were more susceptible to TRAIL than their poorly metastatic primary counterparts. This susceptibility was correlated with the up-regulation of cell surface DR5 and the prominent activation of caspases (caspase-8, -9 and -3) by TRAIL treatment, leading to the inactivation of the DNA-PKcs/Akt pathway through the cleavage of DNA-PKcs and the up-regulation of proapoptotic Bax.

## Results

### High susceptibility of metastatic cancer cells to TRAIL is mediated via DR5 and down-regulation of c-FLIP and Mcl-1

**Figure 1:**
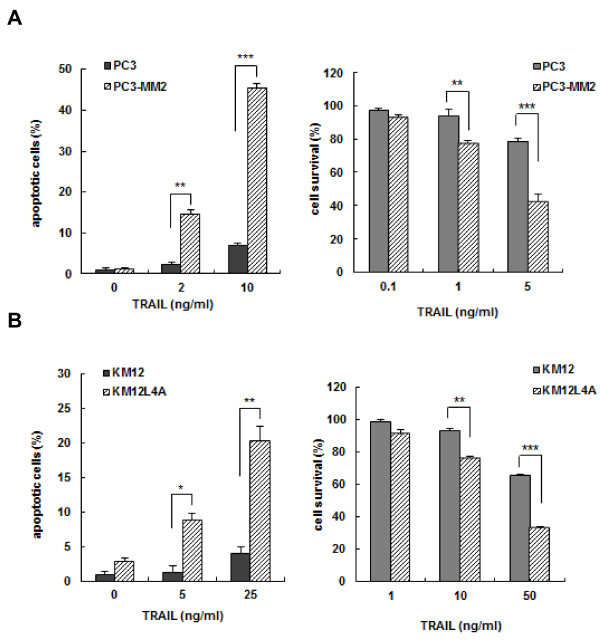
**Differential TRAIL responsiveness of human prostate adenocarcinoma PC3 and colorectal carcinoma KM12 cells and their respective highly metastatic PC3-MM2 and KM12L4A sublines**. PC3 and PC3-MM2 cells **(A) **or KM12 and KM12L4A cells **(B) **were treated with the indicated doses of TRAIL for 6 or 8 h, respectively, and the percentage of apoptotic cells in each cell population was determined using Annexin V staining and flow cytometry (left). In addition, PC3 and PC3-MM2 cells or KM12 and KM12L4A cells were treated with the indicated doses of TRAIL for 96 h, and cell survival (%) was determined using the MTT assay (right). Each value represents the mean ± SE of triplicate determinants. *p < 0.05, **p < 0.01, ***p < 0.001.

To compare the susceptibility of TRAIL-induced apoptosis between of highly metastatic cells and their respective primary cancer cells with low metastatic potential, human prostate adenocarcinoma PC3 and its highly metastatic PC3-MM2 subline were treated with increasing concentrations of TRAIL. Interestingly, PC3-MM2 cells were significantly more sensitive to TRAIL-induced apoptosis and cytotoxicity than PC3 cells (Figure 1A). Similar results were obtained in the highly metastatic colorectal carcinoma KM12L4A cells and its primary KM12 cells. KM12L4A cells were significantly more susceptible to TRAIL-mediated apoptosis and cytotoxicity than KM12 cells (Figure 1B). These results indicated greater susceptibility of highly metastatic cells to TRAIL than their poorly metastatic primary counterparts.

**Figure 2:**
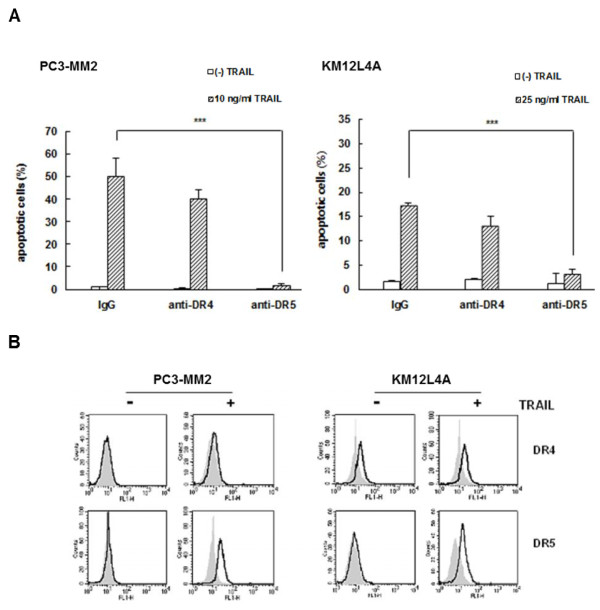
**Effects of neutralizing antibodies to TRAIL receptors on TRAIL-induced apoptosis and expression of TRAIL receptors on the cell surface in PC3-MM2 and KM12L4A cells**. **(A) **PC3-MM2 and KM12L4A cells were pretreated with the anti-DR4 or anti-DR5 antibodies (0.5 μg/ml) for 3 h and then PC3-MM2 and KM12L4A cells were treated with the indicated doses of TRAIL for 6 and 8 h, respectively. Goat IgG was used as the control isotype antibody. Apoptosis was detected by annexin V binding assay. Each value represents the mean ± SE of triplicate determinants. ***p < 0.001. (**B) **PC3-MM2 and KM12L4A cells were treated with 2.5 ng/ml TRAIL for 3 h. Thereafter, cells were stained with control mouse IgG or anti-DR4/DR5 antibody (1:100), and subsequently labeled with FITC-conjugated secondary antibodies (1:200) to determine the surface expression of DR4/DR5. The cell surface expression was measured by a flow cytometer. Shaded and unshaded peaks correspond to control and specific stainings, respectively.

Since it is well known that TRAIL triggers apoptotic signals via two types of death receptors, DR4 and DR5 [7], we examined whether the increased TRAIL-induced apoptosis in highly metastatic cells was mediated via DR4 and/or DR5 death receptors (Figure 2A). We found that treating PC3-MM2 cells with neutralizing antibody to TRAIL receptor DR5 prior to TRAIL treatment inhibited significantly TRAIL-induced apoptosis, while the neutralizing anti-DR4 antibody only slightly inhibited TRAIL-induced apoptosis in the metastatic cells. Similar results were obtained in KM12L4A cells. Therefore, to correlate the inhibitory effects of anti-DR5 neutralizing antibody on TRAIL-induced apoptosis in both PC3-MM2 and KM12 L4A cells with cell surface expression of DR5, we determined the cell surface expression of DR4 and DR5 in the metastatic cells. After treatment with TRAIL, the surface expression of DR5 was significantly up-regulated in both PC3-MM2 and KM12 L4A cells, whereas DR4 expression was not modulated by TRAIL treatment (Figure 2B). These results suggest that the increased susceptibility to TRAIL of highly metastatic cells may be attributed to TRAIL-induced up-regulation of DR5.

**Figure 3:**
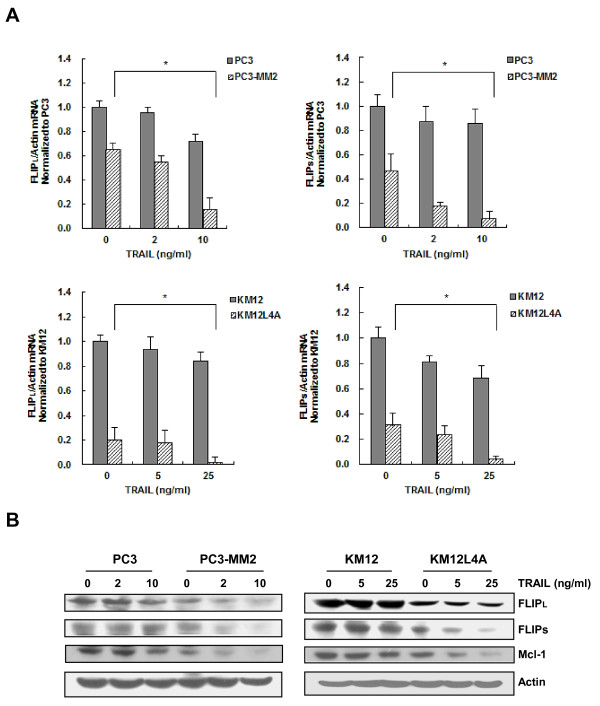
**Down-regulation of c-FLIP_L/S _and Mcl-1 in TRAIL-treated metastatic cancer cells**. PC3 and PC3-MM2 cells were treated with 2 or 10 ng/ml TRAIL for 6 h, and KM12 and KM12L4A cells were treated with 5 or 25 ng/ml TRAIL for 8 h. After TRAIL treatment, the mRNA expression level of c-FLIP_L/S _(FLIP_L/S_) was measured by quantitative real-time RT-PCR **(A)**, and protein levels of FLIP_L/S _and Mcl-1 were measured by Western blot analysis **(B)**, respectively. β-Actin (Actin) was used as a loading control.

Since the expression of cellular FADD-like interleukin-1β-converting enzyme (FLICE)-inhibitory proteins (c-FLIP_L _and c-FLIP_S_) potently controls the susceptibility of cancer cells to TRAIL-induced apoptosis, we assessed the mRNA and protein levels of c-FLIP_L/S _in the metastatic cells treated with or without TRAIL using quantitative real-time RT-PCR and Western blot analysis, respectively. The results of quantitative real-time RT-PCR showed that basal mRNA levels of c-FLIP_L/S _were significantly down-regulated in PC3-MM2 and KM12 L4A cells compared with PC3 and KM12 cells, respectively, and after treatment with TRAIL the mRNA levels of c-FLIP_L/S _were significantly decreased in PC3-MM2 and KM12L4A cells (Figure 3A). These changes in mRNA levels of c-FLIP_L/S _were followed by corresponding changes in their protein levels (Figure 3B). We also compared the expression level of myeloid cell leukemia sequence 1 (Mcl-1) protein between two pairs of primary and their highly metastatic cells, because Mcl-1 as well as c-FLIP is also the main determinant of acquired TRAIL resistance [17]. Like c-FLIP_L/S_, the basal level of Mcl-1 was lower in highly metastatic PC3-MM2 and KM12L4A cells compared with their respective primary PC3 and KM12 cells, and the down-regulation of Mcl-1 by treatment with TRAIL was more sensitive in highly metastatic PC3-MM2 and KM12L4A cells compared with their respective primary PC3 and KM12 cells (Figure 3B). Therefore, our data indicated that high susceptibility to TRAIL of metastatic cancer cells is associated with up-regulation of DR5 and concurrent down-regulation of c-FLIP and Mcl-1 by TRAIL treatment.

### Increased expression of c-Myc in metastatic cancer cells is associated with the increased susceptibility to TRAIL

**Figure 4:**
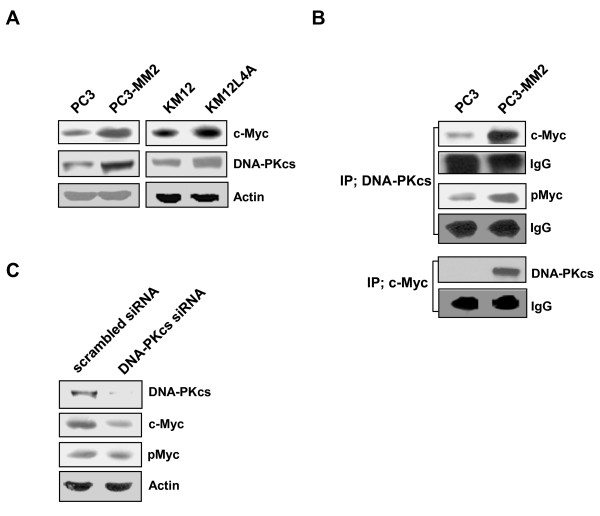
**Over-expression of c-Myc and the cross-talk between DNA-PKcs and c-Myc in metastatic cancer cells**. **(A) **The levels of c-Myc and DNA-PKcs proteins in PC3 and PC3-MM2 cells (left) and KM12 and KM12L4A cells (right) were determined using Western blot analysis. **(B) **Immunoprecipitates (IP) of the DNA-PKcs or c-Myc antibody from PC3 and PC3-MM2 cells were subjected to Western blot analysis with c-Myc, phospho-c-Myc (pMyc) or DNA-PKcs antibody to determine the interaction between DNA-PKcs and c-Myc. IgG was used as an internal control for the immunoprecipitation. **(C) **PC3 cells were transfected with siRNA against DNA-PKcs or scrambled siRNA as a control. After 48 h, the cell lysates of the transfectants were subjected to Western blot analysis with c-Myc or pMyc antibody.

It has been known that c-Myc renders cancer cells sensitive to TRAIL-induced apoptosis by the up-regulation of DR5, the activation of caspase-8, and the down-regulation of c-FLIP and Mcl-1 [13,18,19]. Since the metastatic PC3-MM2 cells showed the down-regulation of c-FLIP and Mcl-1, we determined the level of c-Myc expression in metastatic cancer cells. PC3-MM2 and KM12L4A cells showed significantly higher basal level of c-Myc than their primary PC3 and KM12 cells (Figure 4A). Since DNA-PK can phosphorylate c-Myc at several serine residues *in vitro *[20] and DNA-PKcs modulates the stability of c-Myc [15], we compared the level of DNA-PKcs between the primary and their cognate metastatic cancer cells. The level of DNA-PKcs was higher in metastatic PC3-MM2 and KM12L4A cells than in their primary cells (Figure 4A). These results were followed by the increased interaction between c-Myc and DNA-PKcs in PC3-MM2 cells compared with PC3 cells in immunoprecipitation assay (Figure 4B) and the down-regulated levels of c-Myc and phospho-c-Myc (pMyc) after knock-down of DNA-PKcs with specific siRNA in PC3 cells (Figure 4C). These results suggest that the increased level of DNA-PKcs may be contributory to the over-expression of c-Myc protein in metastatic cancer cells.

**Figure 5:**
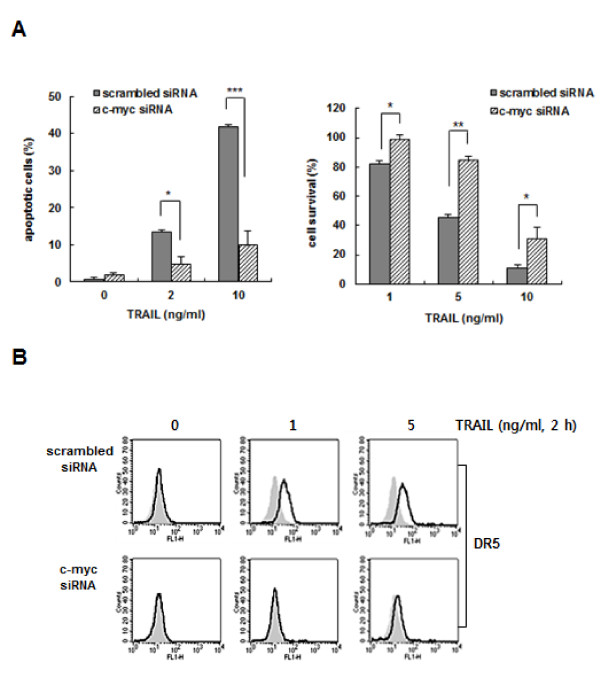
**Targeted disruption of the c-Myc gene reduced the responsiveness to TRAIL and the expression of DR5 in PC3-MM2 cells**. **(A) **PC3-MM2 cells were transfected with siRNA against c-Myc or scrambled siRNA as a control. After 48 h, the transfectants were treated with TRAIL (2 or 10 ng/ml) for 6 h, and the percentage of apoptotic cells was determined using Annexin V staining and flow cytometry (left). In addition, the transfectants were treated with graded single doses of TRAIL (1~10 ng/ml), and percentage of cell survival was determined after 96 h incubation using the MTT assay (right). Data represent the mean ± SE of triplicate experiments. *p < 0.05, **p < 0.01, ***p < 0.001. **(B) **The transfected cells incubated with anti-DR5 antibody (1:100), and subsequently labeled with FITC-conjugated secondary antibodies (1:200) to determine the surface expression of DR5. Mouse IgG was also used as an isotype control. The shaded and unshaded peaks correspond to control and specific staining, respectively.

To examine the effect of c-Myc on the sensitivity of the metastatic cells to TRAIL, PC3-MM2 cells were transfected with c-Myc siRNA or a scrambled siRNA. TRAIL-induced cell death was decreased by transfection of c-Myc siRNA in PC3-MM2 cells (Figure 5A). Moreover, siRNA against c-Myc prevented the TRAIL-induced cell surface expression of DR5 (Figure 5B). Therefore, these results suggest that the increased level of c-Myc contributes to the hypersensitivity of metastatic cells to TRAIL, possibly in part due to TRAIL-induced surface expression of DR5.

### Activation of pro-caspases is accelerated after treatment with TRAIL in metastatic cancer cells over-expressing c-Myc

**Figure 6:**
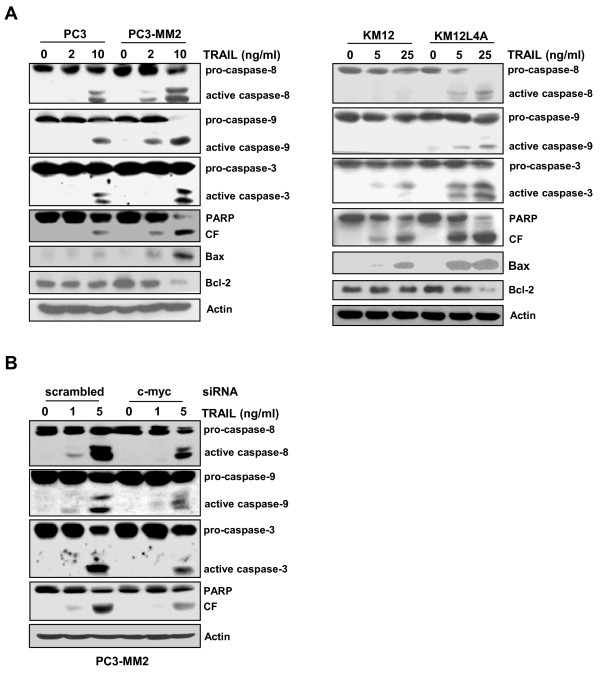
**Increased activation of pro-caspases in TRAIL-treated metastatic cancer cells**. **(A) **The cell lysates obtained from PC3 and PC3-MM2 cells treated with 2 or 10 ng/ml TRAIL for 6 h (left) or KM12 and KM12L4A cells treated with 5 or 25 ng/ml TRAIL for 8 h (right) were subjected to Western blot analysis to monitor the levels of caspase-8, -9 and -3, Bax and Bcl-2. The levels of PARP and its cleavage fragment (CF) in TRAIL-treated cells were also determined. **(B) **PC3-MM2 cells were transfected with siRNA against c-myc or scrambled siRNA as a control. After 48 h, the transfectants were treated with TRAIL (1 or 5 ng/ml) for 6 h and were subjected to Western blot analysis to monitor the change in activities of caspases and PARP. Actin was used as a loading control.

c-Myc acts as a gatekeeper of the caspase feedback amplification loop and c-Myc-mediated priming of the mitochondrial pathway enables weak caspase-8 signals to activate effector caspases and establish a death executing caspase feedback amplification loop [12]. Therefore, we determined if the increased susceptibility of metastatic cancer cells over-expressing c-Myc to TRAIL-induced apoptosis was accompanied by the increased activation of caspases (Figure 6A). Consistent with the hypersensitive response to TRAIL-induced cell death of metastatic cancer cells over-expressing c-Myc, the TRAIL-induced cleavage of procaspase-8, which is an initiator caspase linked to the receptor-mediated apoptotic pathway, was remarkably increased in the metastatic PC3-MM2 and KM12L4A cells as compared to the PC3 and KM12 cells, respectively. The proteolytic processing of caspase-9, which has been linked to the mitochondrial death pathway, was significantly induced in the metastatic cells following treatment of with TRAIL compared to their primary cells. In addition, the cleavage of procaspase-3, an executioner caspase, and PARP, a hallmark of caspase-3 activation, was profoundly increased in metastatic cells compared with their primary counterparts. We also investigated whether the modulation of proapoptotic Bax and antiapoptotic Bcl-2 proteins was involved in the TRAIL-induced apoptosis of the metastatic cells. The expression of Bax and Bcl-2 was up-regulated and down-regulated following treatment with TRAIL, respectively, in the PC3-MM2 and KM12L4A cells compared to their corresponding counterparts, consistent with the activation status of pro-caspase-9 in these cells. These results were followed by reduction of TRAIL-induced caspase activation and PARP cleavage by depletion of c-Myc with c-Myc siRNA in PC3-MM2 cells (Figure 6B). Therefore, these data demonstrated that the high susceptibility of metastatic cells to TRAIL was at least in part due to the increased expression of c-Myc, suggesting that TRAIL effectively may induce caspase-dependent apoptosis in metastatic cells over-expressing c-Myc.

### The amplified TRAIL-induced caspase activation by overexpressed c-Myc counteract the anti-apoptotic activity of DNA-PKcs by increasing proteolytic cleavage in the metastatic cancer cells

**Figure 7:**
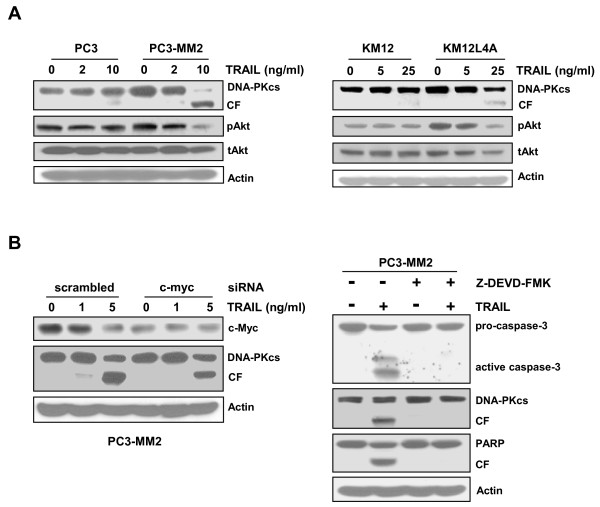
**Down-regulation of DNA-PKcs/pAkt in TRAIL-treated metastatic cancer cells and prevention of caspase-3 dependent DNA-PKcs cleavage by suppression of c-Myc in the cells**. **(A) **The cell lysates obtained from PC3 and PC3-MM2 cells (left) treated with TRAIL (2 or 10 ng/ml for 6 h) or KM12 and KM12L4A cells (right) treated with TRAIL (5 or 25 ng/ml for 8 h) were subjected to Western blot analysis to monitor the levels of DNA-PKcs and its CF, phosphorylated Akt Ser473 (pAkt) and total Akt (tAkt). Actin was used as a loading control. **(B) **PC3-MM2 cells were transfected with siRNA against c-myc or scrambled siRNA as a control. After 48 h, the transfectants were treated with TRAIL (1 or 5 ng/ml) for 6 h and were subjected to Western blot analysis to monitor the changed levels of c-Myc and DNA-PKcs (left). The cell lysates of PC3-MM2 cells treated with TRAIL (5 ng/ml) for 4 h were subjected to Western blot analysis to monitor levels of caspase-3, DNA-PKcs, PARP and their CFs. Some samples were pretreated with 50 μM Z-DEVD-FMK, a specific caspase-3 inhibitor, for 2 h (right).

It has been known that a constitutively active Akt is an important regulator of TRAIL sensitivity [21,22], and inhibition of Akt activation by its pharmacological inhibitor or knockdown of its expression by siRNA sensitizes TRAIL-resistant cells to TRAIL [23,24]. The activation of Akt by phosphorylation of Ser-473 is mediated by DNA-PKcs [25]. However, the metastatic cancer cells were sensitive to TRAIL, despite that the metastatic cells have higher level of DNA-PKcs compared with their primary cells, as shown above. Therefore, we determined the levels of DNA-PKcs and pAkt in the metastatic cells after treatment with TRAIL (Figure 7A). In metastatic PC3-MM2 and KM12L4A cells, DNA-PKcs was cleaved and consequently the level of DNA-PKcs was decreased after exposure to TRAIL and this result was accompanied with decrease of pAkt level, whereas the levels of DNA-PKcs and pAkt were maintained in the primary PC3 and KM12 cells after exposure to TRAIL.

Since c-Myc could control the TRAIL sensitivity, and DNA-PKcs is a substrate of caspase-3 [26], we determined whether depletion of c-Myc could block degradation of DNA-PKcs. When PC3-MM2 cells were transfected with c-Myc siRNA, TRAIL-induced cleavage of DNA-PKcs was reduced compared with transfection with scrambled siRNA. These results were followed by prevention of cleavage of DNA-PKcs as well as PARP in the TRAIL-treated cells by pretreatment with Z-DEVD-FMK, a caspase-3-specific inhibitor (Figure 7B). These results suggest that the amplified TRAIL-induced caspase activation by over-expressed c-Myc may curtail the anti-apoptotic activity of DNA-PKcs by increasing its proteolytic cleavage in the metastatic cancer cells.

### Suppression of DNA-PKcs is associated with hypersensitivity to TRAIL-induced cytotoxicity

**Figure 8:**
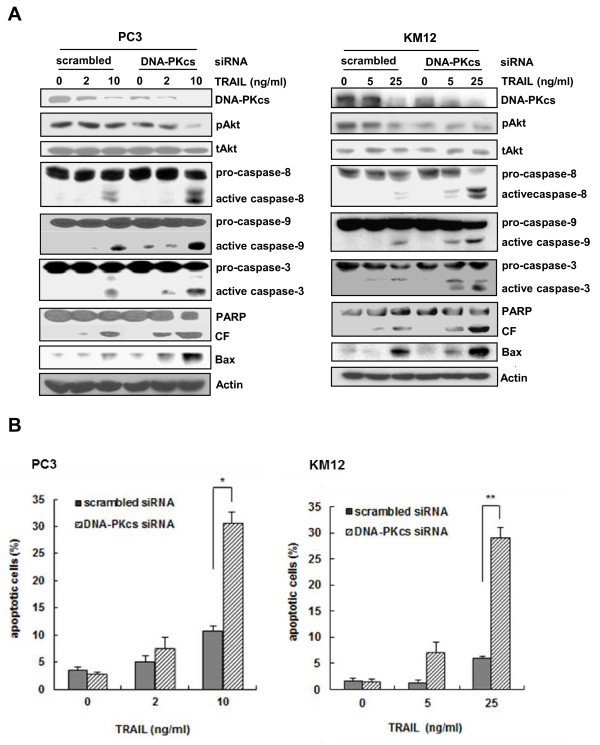
**Suppression of DNA-PKcs leads to activation of caspase and Bax through the inactivation of the DNA-PKcs/Akt signaling pathway and enhances responsiveness to TRAIL**. **(A) **PC3 (left) and KM12 cells (right) were transfected with siRNA against DNA-PKcs or scrambled siRNA as a control. After 48 h, the transfected PC3 and KM12 cells treated with the indicated doses of TRAIL for 6 and 8 h, respectively, and were subjected to Western blot analysis to monitor the levels of DNA-PKcs, pAkt, tAkt, caspases (caspase-8, -9, and -3) and Bax. The levels of PARP and its cleavage fragment (CF) in the transfectants were also determined. Actin was used as a loading control. **(B) **PC3 (left) and KM12 cells (right) were transfected with siRNA against DNA-PKcs or scrambled siRNA as a control. After 48 h, the transfected cells were treated with the indicated doses of TRAIL for 6 and 8 h, respectively. Thereafter, the percentage of apoptotic cells was determined using Annexin V staining and flow cytometry (right). Each value represents the mean ± SE of triplicate determinants. *p < 0.05, **p < 0.01.

To investigate the direct role of DNA-PKcs in the susceptibility of the metastatic cells to TRAIL, we used siRNA to knockdown DNA-PKcs expression and determined its effect on TRAIL sensitivity. After transfection of PC3 or KM12 cells with siRNA against DNA-PKcs or scrambled siRNA, the expression of DNA-PKcs was efficiently suppressed by DNA-PKcs siRNA as compared to the control cells and its expression was further decreased by TRAIL treatment (Figure 8A). This result was followed by the hypersensitivity to TRAIL-induced reduction of DNA-PKcs/pAkt levels, activation of caspases, PARP cleavage, and up-regulation of Bax in PC3 and KM12 cells after transfection with DNA-PKcs siRNA as compared to the cells transfected with scrambled siRNA. Furthermore, the knockdown of DNA-PKcs with specific siRNA significantly increased TRAIL-induced apoptosis in PC3 and KM12 cells (Figure 8B).

**Figure 9:**
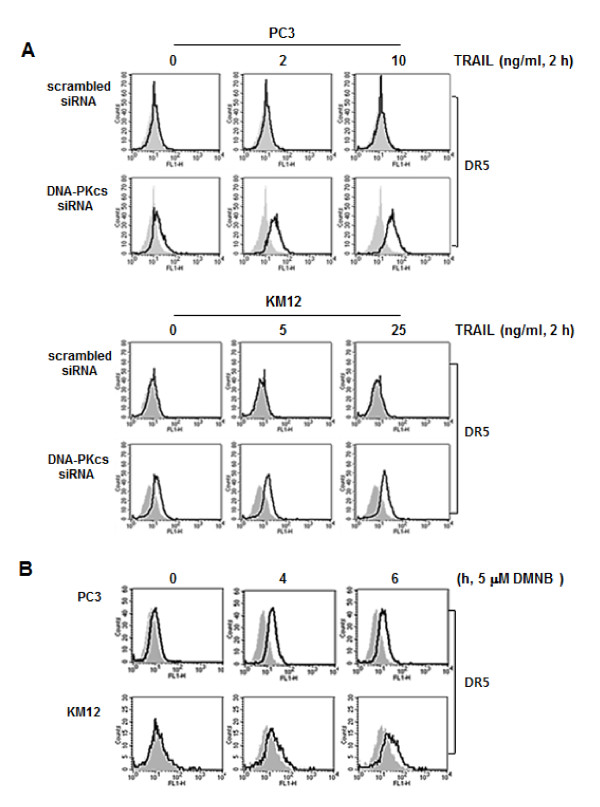
**Suppression of DNA-PKcs leads to an increase of cell surface expression of DR5**. **(A) **PC3 and KM12 cells were transfected with siRNA against DNA-PKcs or scrambled siRNA as a control. After 48 h, the transfected cells were treated with the indicated doses of TRAIL for 2 h. Thereafter, the transfected cells were incubated with anti-DR5 antibody (1:100), and subsequently labeled with FITC-conjugated secondary antibodies (1:200) to determine the surface expression of DR5. **(B) **PC3 (upper) and KM12 cells (lower) were treated with 5 μM DMNB for 4 and 6 h, and then incubated with an anti-DR5 antibody (1:100), and subsequently labeled with FITC-conjugated secondary antibodies (1:200) to determine the surface expression of DR5. Mouse IgG was used as an isotype control. The shaded and unshaded peaks correspond to control and specific staining, respectively.

Since knockdown of c-Myc suppressed the TRAIL-induced cleavage of DNA-PKcs and knockdown of DNA-PKcs increased TRAIL-induced activation of caspases, c-Myc overexpressed in the metastatic cancers may increase TRAIL-induced cleavage of DNA-PKcs and consequently caspase-mediated apoptosis. We then determined whether the knockdown of DNA-PKcs leads to the enhancement of TRAIL sensitivity via the up-regulation of the cell surface expression of death receptors. The suppression of DNA-PKcs with specific siRNA in the PC3 and KM12 cells resulted in an increase in the cell surface expression of DR5 (Figure 9A), but not DR4 (data not shown), and this up-regulation was increased further by TRAIL treatment. Moreover, we also showed that the cell surface expression of DR5 in PC3 and KM12 cells was induced by treatment with 4,5-dimethoxy-2-nitrobenzaldehyde (DMNB) [27], a specific inhibitor of DNA-PK (Figure 9B). These results indicate that the inhibition of the DNA-PKcs/Akt signaling pathway may contribute to the sensitization of PC3 or KM12 cells to TRAIL-induced apoptosis through the up-regulation of DR5 cell surface expression and the activation of caspase cascade.

### Combination of DMNB and TRAIL renders PC3 and KM12 cells highly susceptible to TRAIL-induced apoptosis

**Figure 10:**
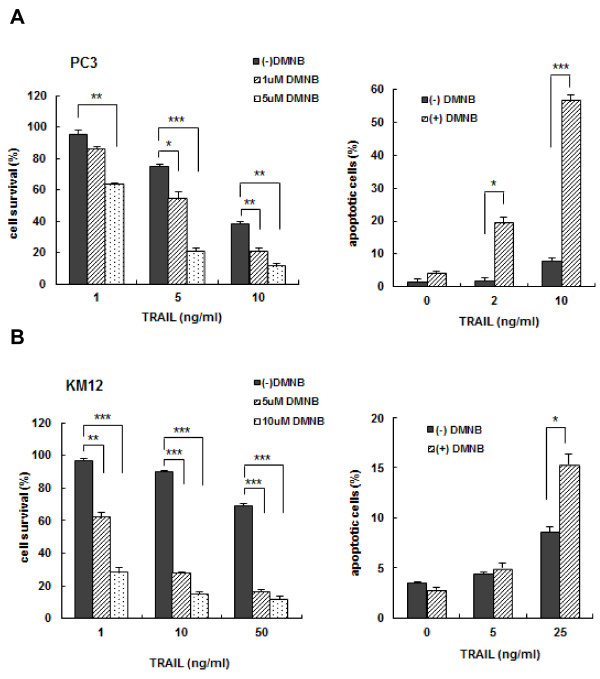
**Effect of DMNB on TRAIL-induced cytotoxicity and apoptosis**. PC3 **(A) **and KM12 **(B) **cells were treated with the indicated doses of TRAIL in the presence or absence of DMNB (1, 5, or 10 μM), and cell survival was determined after 96 h of incubation using the MTT assay (left). PC3 and KM12 cells were treated with the indicated doses of TRAIL in the presence or absence of 5 μM DMNB for 6 or 8 h, respectively. Thereafter, the percentage of apoptotic cells was determined using Annexin V staining and flow cytometry (right). Each point is the average of triplicate determinants. *p < 0.05, **p < 0.01, ***p < 0.001.

**Figure 11:**
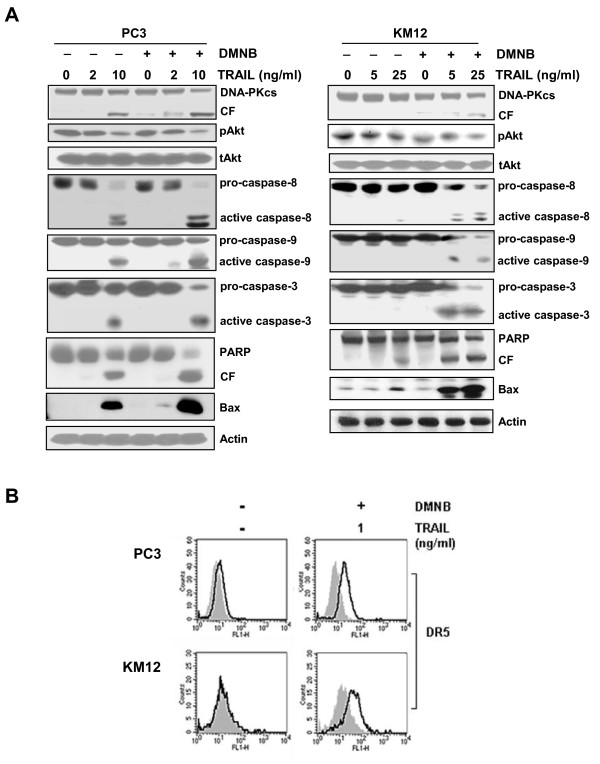
**Combination effects of DMNB and TRAIL on DNA-PKcs/Akt molecules and caspase activity**. **(A) **PC3 cells (left) and KM12 cells (right) were treated with the indicated doses of TRAIL in the presence or absence of 5 μM DMNB for 6 or 8 h, respectively, and assessed using Western blot analysis to monitor the protein levels of DNA-PKcs, pAkt and tAkt. The activation of caspases and Bax and the cleavage of PARP in cells treated with TRAIL and/or DMNB were determined by Western blot analysis. Actin was used as a loading control. **(B) **PC3 and KM12 cells were treated with 1 ng/ml TRAIL in the presence or absence of 5 μM DMNB for 2 h, and then incubated with an anti-DR5 antibody (1:100), and subsequently labeled with FITC-conjugated secondary antibodies (1:200) to determine the surface expression of DR5. Mouse IgG was used as an isotype control. The shaded and unshaded peaks correspond to control and specific staining, respectively.

Since the suppression of DNA-PKcs level with siRNA induced the up-regulation of DR5 and the activation of caspases, we determined whether DMNB could potentiate TRAIL-induced cytotoxicity and apoptosis in TRAIL-resistant PC3 and KM12 cells and function as a TRAIL sensitizer. DMNB in combination with TRAIL sensitized PC3 cells (Figure 10A) and KM12 cells (Figure 10B) to TRAIL-induced cytotoxicity and apoptosis in a dose-dependent manner. We next examined whether the enhanced susceptibility of PC3 and KM12 cells to TRAIL following DMNB treatment was associated with caspase activation and the up-regulation of Bax through the inactivation of the DNA-PKcs/Akt signaling (Figure 11A). Co-treatment of PC3 or KM12 cells with TRAIL and DMNB resulted in a decrease in the levels of both DNA-PKcs and pAkt when compared to cells treated with TRAIL alone. The combination of DMNB and TRAIL was more effective for the activation of caspases, the inactivation of the DNA-PKcs/Akt signaling pathway, PARP cleavage, and the up-regulation of Bax than the treatment with TRAIL alone. In addition, combined treatment of DMNB and TRAIL increased surface expression of DR5 in both PC3 and KM12 cells, which did not respond to TRAIL alone (Figure 11B). These results suggest that the inactivation of the DNA-PKcs/Akt signaling pathway with siRNA or small molecules may be a useful strategy to increase the susceptibility of TRAIL-resistant solid tumor cells to TRAIL-induced cell death.

## Discussion

It is unclear why some cells are sensitive to TRAIL-induced apoptotic stimuli, whereas other cell types survive after exposure to TRAIL. Therefore, it is necessary to characterize the molecular mechanisms underlying this apoptotic sensitivity. Furthermore, the molecular determinants regulating TRAIL sensitivity in metastatic cancer cells are still poorly understood. In this study, we demonstrated the differential TRAIL responsiveness of primary human prostate adenocarcinoma PC3 and colorectal carcinoma KM12 cells and their respective highly metastatic PC3-MM2 and KM12L4A sublines. Interestingly, PC3-MM2 and KM12L4A cells were more susceptible to TRAIL than their primary counterparts. We found that DR5 could be the major receptor involved in TRAIL-mediated apoptosis of these highly metastatic cells. Previously, we and other group reported that the expression of DR5 in MDR cells was up-regulated following TRAIL treatment [28,29]. In case of our results, cell surface expression of DR5, but not DR4, in PC3-MM2 and KM12L4A cells was up-regulated after TRAIL treatment whereas DR4/DR5 expression in primary PC3 and KM12 cells was not modulated by TRAIL treatment (data not shown). Moreover, pre-treating PC3-MM2 and KM12L4A cells with a neutralizing antibody against the DR5 receptor strongly reduced the degree of apoptosis induced by TRAIL. However, DR4 neutralizing antibody did not significantly affect TRAIL-triggered apoptosis in these highly metastatic cells, indicating that TRAIL induced apoptosis occurs preferentially via DR5, and therefore, DR5 could be the major receptor involved in increased susceptibility of metastatic cells to TRAIL. We also found that the levels of c-FLIP_L/S _and Mcl-1, the major factors for resistance to TRAIL-induced apoptosis, were significantly decreased in PC3-MM2 and KM12L4A cells compared to their primary counterparts. Therefore, our data suggest that high susceptibility to TRAIL of metastatic cancer cells is associated with up-regulation of DR5 and concurrent down-regulation of c-FLIP_L/S _and Mcl-1.

It has been reported that TRAIL-resistant cells can be resensitized by c-Myc, which induces DR5 expression, and represses the transcription of both c-FLIP and Mcl-1 [30]. Consistently, in our data the expression of c-Myc was significantly increased in PC3-MM2 and KM12L4A cells with high metastatic potency compared to their primary cells. c-Myc is deregulated in a wide range of human cancers, including breast, colon, cervical and small-cell lung carcinomas, osteosarcomas, glioblastomas, melanoma and myeloid leukaemias, and c-Myc over-expression is frequently correlated with aggressive metastasis, poor differentiation and poor prognosis [31]. Paradoxically, in addition to promoting proliferation, c-Myc inhibits survival signals, resulting in apoptosis, if the apoptotic signal is sufficiently strong [11]. On the other hand, activation of c-Myc renders primary and non-transformed cells sensitive to TNF-α, CD95L, and TRAIL-induced apoptosis [13,18,19,32,33]. It is known that c-Myc can alter the expression of several key players of the death receptor pathway, such as up-regulation of DR5 [13] and down-regulation of c-FLIP [19], promoting the activation of death receptors and DISC and, thus, renders cells sensitive to TRAIL-induced apoptosis [31]. In addition, it was demonstrated that c-Myc suppresses *Mcl-1 *transcription, and is constitutively bound to the *Mcl-1 *promoter [18]. Therefore, our results suggest that increased sensitivity to TRAIL in metastatic cancer cells may be in part due to increased expression of c-Myc in the cells, which is associated with up-regulation of DR5 cell surface expression and down-regulation of c-FLIP and Mcl-1. In addition to enhancement of death-receptor signaling, c-Myc also amplifies the death signal at the mitochondria for synergistic induction of apoptosis by activating Bak, enabling TRAIL to fully activate the caspase machinery in human cells [10,12]. We found that after treatment with TRAIL activity of caspases including caspase-8, -9, and -3 were higher, and up-regulation of Bax and down-regulation of Bcl-2 were more significant in PC3-MM2 and KM12L4A cells than in their primary cells. Since c-FLIP_L _and Mcl-1 were known as substrates of caspases [34,35], it could be possible that TRAIL-induced potent activation of caspases in metastatic cancer cells led to accelerated down-regulation of c-FLIP_L _and Mcl-1. Therefore, these increased proapoptotic responses to TRAIL in the highly metastatic cancer cells might be attributed to the increased level of c-Myc.

Moreover, PC3-MM2 and KM12L4A cells showed the enhanced constitutive expression of DNA-PKcs as well as c-Myc compared to the corresponding primary cells. DNA-PK, a complex consisting of the regulatory subunits Ku70/80 and the catalytic subunit DNA-PKcs, plays a central role in the repair of DNA double-strand breaks (DSB). Over-expression of DNA-PKcs was reported in various human tumors [36-38], and the activity and protein/mRNA levels of DNA-PKcs were significantly higher in tumor tissues than in normal tissues [39]. Previously we demonstrated that the DNA-PK activity is remarkably increased in metastatic cancer cells [14]. Although DNA-PKcs was primarily defined as a component of the DNA DSB repair complex, DNA-PKcs is implicated, directly and indirectly, in various cellular metabolic processes, since DNA-PKcs may be able to phosphorylate the oncoproteins c-Myc, c-fos, and c-abl [40]. DNA-PKcs activity may contribute to the overexpression of c-Myc, probably via its critical role in maintaining the stability of c-Myc [15,40]. DNA-PKcs also activates Akt via phosphorylation of Ser473, which in turn inactivates GSK-3β via phosphorylation of Ser9, resulting in the stabilization of c-Myc [15]. We showed a prominent interaction between DNA-PKcs and c-Myc and an increased level of phosphorylated c-Myc levels in PC3-MM2 cells compared to PC3 cells, suggesting the possibility that the overexpressed DNA-PKcs might contribute to the overexpression c-Myc in metastatic cancer cells via its stabilization. As previously described, c-Myc can participate in cell death as a pro-apoptotic factor [10,12]. In our experiment, we demonstrated that siRNA-mediated depletion of c-Myc in metastatic cancer cells suppressed TRAIL-induced up-regulation of DR5 and activation of caspase, and these phenomena could be associated with decreased cytotoxic effect of TRAIL on PC3-MM2 and KM12L4A cells.

It is known that the inhibition of Akt in gliomas enhances their susceptibility to TRAIL [41] and TRAIL down-regulates Akt levels by caspase-dependent degradation [42]. In addition, DNA-PKcs, an upstream regulator of Akt, is cleaved by caspase-3 and, thus, the inactivation of these molecules occurs during apoptosis [43,44]. Our data showed that the cleavage of DNA-PKcs and the subsequent reduction of DNA-PKcs and pAkt levels occurred in the PC3-MM2 and KM12L4A cells after TRAIL treatment, but not in their primary counterparts, even though the metastatic cells exhibited higher basal levels of DNA-PKcs and pAkt than their primary counterparts. This TRAIL-induced suppression of DNA-PKcs/Akt signaling pathway in metastatic cancer cells seemed to be at least in part due to overexpression of c-Myc, since it was prevented by knockdown of c-Myc. Therefore, our results suggest that the amplified TRAIL-induced caspase activation by overexpressed c-Myc may suppress the anti-apoptotic activity of DNA-PKcs by increasing its proteolytic cleavage and consequently promote apoptosis in the metastatic cancer cells. Finally, we found that suppression of DNA-PKcs by siRNA and DNMB significantly increased TRAIL-induced growth inhibition and apoptosis in TRAIL-insensitive PC3 or KM12 cells, which was accompanied by the inhibition of Akt S473 phosphorylation, the activation of caspases (caspase-8, -9, and -3), and the up-regulation of the cell surface expression of DR5. Indeed, it has been reported that inhibitors of DNA-PK have a great potential to enhance the efficacy of chemotherapy and radiotherapy [45-47].

## Conclusions

This study showed the positive relationship between c-Myc expression in highly metastatic human prostate and colon cancer cells and susceptibility to TRAIL-induced apoptosis, which indicated that TRAIL might be used as an effective therapeutic modality for advanced metastatic cancers overexpressing c-Myc. In addition, DMNB, a specific inhibitor of DNA-PK, potentiated TRAIL-induced cytotoxicity and apoptosis in relatively TRAIL-insensitive KM12 and PC3 cells and therefore functioned as a TRAIL sensitizer, which might be clinically useful for the treatment of relatively TRAIL-insensitive human cancers.

## Methods

### Cell culture and Reagents

The poorly metastatic primary human colorectal carcinoma KM12 cells were originally derived from a Dukes' B2 colon cancer. The highly liver-metastatic KM12L4A cells were derived from KM12 cells. Human androgen-independent PC3 prostate adenocarcinoma cells and PC3-MM2 cells, a variant of PC3 selected for their highly metastatic potential were used in this study. These four cell lines were provided by Professor IJ Fidler (MD Anderson, TX, USA). The cells were maintained in culture (5% CO_2 _and 95% at 37°C) as adherent monolayers in DMEM supplemented with 10% fetal bovine serum (FBS), sodium pyruvate, nonessential amino acids, L-glutamine, and vitamin solution. The recombinant human soluble TRAIL was obtained from R&D Systems (Minneapolis, MN, USA). 4,5-dimethoxy-2-nitrobenzaldehyde (DMNB) was purchased from Merck KgaA (Darmstadt, Germany).

### Cell Proliferation Assay (MTT assay)

Cell proliferation was measured by counting viable cells by using the 3-(4,5-dimethylthiazol-2-yl)-2,5-diphenyltetrazolium bromide (MTT; Sigma Chemical Company, St. Louis, MO, USA) colorimetric dye-reduction method. Exponentially growing cells (5 × 10^3 ^cells/well) were plated in a 96-well plate and incubated in growth medium treated with the indicated concentration of TRAIL and/or DMNB at 37°C. After 96 h, the medium was aspirated using centrifugation and MTT-formazan crystals solubilized in 100 μl DMSO. The optical density of each sample at 570 nm was measured using ELISA reader. The optical density of the medium was proportional to the number of viable cells. Inhibition of proliferation was evaluated as a percentage of control growth (no drug in the medium). All experiments were repeated in at least two experiments in triplicate.

### Flow cytometry analysis

Cells (5 × 10^5 ^cells/well) treated with or without TRAIL (or DMNB) were centrifuged at 500 × g, washed with phosphate-buffered saline (PBS), and resuspended in 500 μl PBS. The cells were then incubated with 5 μl mouse IgG, or anti-DR4 or -DR5 monoclonal mouse antibody (1:100; R&D Systems, Minneapolis, MN, USA) for 2 h. After washing with PBS, FITC-conjugated rabbit anti-mouse IgG (1:200; Sigma Chemical Co., St. Louis, MO, USA) was added to the cell suspension and incubated for 2 h on ice followed by washing with PBS. After rinsing, the samples were analyzed by flow cytometry using a FACSCalibur flow cytometer (Becton Dickinson, San Jose, CA, USA). The data were analyzed using the CellQuest program.

To determine whether TRAIL-induced apoptosis occurs through the death receptors, cells were pretreated with the TRAIL-R1 (anti-DR4) or TRAIL-R2 (anti-DR5) antibody (0.5 μg/ml, R & D Systems, Minneapolis, MN) 3 h before treatment with 2 and 10 ng/ml TRAIL for 6 h. In control experiments, cells were treated normal goat IgG (Sigma-Aldrich Corp., St Louis, MO, USA) before TRAIL treatment. Apoptosis was the measured by annexin V assay.

### Quantitative real-time RT-PCR

Quantitative analysis of FLIP_L/S _mRNA levels was performed by the SYBR Green real time PCR method using 2X Power SYBR^® ^Green PCR Master Mix (Applied Biosystems, CA, USA) in a Bio-Rad iCycler & iQ Real-time PCR systems (Bio-Rad, Hercules, CA, USA). An increase in the fluorescence of the reporter dye, SYBR green, during quantitative real-time RT-PCR is due to the SYBR green binding to double-stranded DNA. The sequences of forward and reverse primers for FLIP_L_, FLIP_S _and β-actin (as loading control) are the following: FLIP_L _(forward) 5'-TTCCAGGCTTTCGGTTTCTT-3' and (reverse) 5'-GTCCGAAACAAGGTGAGGGT-3'; FLIP_S _(forward) 5'-ACCCTCACCTTGTTTCGGAC-3' and (reverse) 5'-CTTTTGGATTGCTGCTTGGA-3'; and β-actin (forward) 5'-CAGAGCAAGAGAGGCATCCT-3' and (reverse) 5'-TTGAAGGTCTC AAACATGAT-3'. After initial denaturation step at 95°C for 10 min, each amplification step was repeated 60 cycles with the following condition: denaturation at 95°C for 30 sec, annealing at 55°C for 60 sec and extension at 60°C for 60 sec. Each sample was tested in triplicate, and gene expression levels were normalized to β-actin mRNA.

### Western blot and immunoprecipitation analysis

Protein samples were separated by SDS-PAGE and blotted onto a nitrocellulose membrane (Hybond-ECL; GE Healthcare, Buckinghamshire, UK). The membrane was incubated with antibody as specified, followed by a secondary antibody conjugated with horseradish peroxidase. Specific antigen-antibody complexes were detected using enhanced chemiluminescence (PerkinElmer Life Sciences, MA, USA). Western blot analysis was performed with the following antibodies: Bax, caspase-3, PARP and Bcl-2, (Santa Cruz Biotechnology, CA, USA), Akt, phospho-Akt (Ser 473), caspase-8, caspase-9 (Cell signaling Technology, MA, USA), DNA-PKcs (Thermo Fisher Scientific, CA, USA), c-Myc, phospho-c-Myc (pMyc, Epitomics, CA, USA), and β-actin (Sigma-Aldrich, St. Louis, MO, USA). Secondary antibodies were obtained from GE Healthcare (Buckinghamshire, UK). For immunoprecipitation, whole cell or nuclear extracts from PC3 and PC3-MM2 cells were incubated with anti-DNA-PKcs or c-Myc antibody overnight at 4°C, and then protein G-Sepharose beads (Sigma-Aldrich Corp., St Louis, MO, USA) were added and constantly mixed for 4 h. The beads were collected by centrifugation for 5 min at 15,000 × g and 4°C, and washed 3 times with cold extraction buffer. The beads with immune complexes were boiled and electrophoresed on 8% SDS-PAGE gels and analyzed by Western blotting using antibodies against c-Myc, pMyc or DNA-PKcs.

### siRNA Transfection

The DNA-PKcs (5'-CAGUCUUAGUCCGGAUCAUdTdT-3'), c-Myc (5'-GACAGUGUCAGAGUCCUGAdTdT-3'), and control scrambled (5'-CUUCCCGAAAACUUGAGACdTdT-3') siRNAs were used in this study. Cells were transfected with 0.1 μM siRNA for 48 h using oligofectamine according to the manufacturer's instructions (Invitrogen, Carlsbad, CA, USA). In brief, the siRNA/oligofectamine complex was added to cells (2 × 10^5 ^cells/well) that were seeded in 6-well plate. The cells were incubated for 4 h at 37°C in serum free DMEM medium and then FBS was added. After 48 h, the cells were treated with TRAIL and collected for Western blot analysis to determine the levels of DNA-PKcs, c-Myc, and the indicated proteins.

### Apoptosis assay

Untransfected control cells or transfected with the various siRNAs cells (2 × 10^5 ^cells/ml) were treated with or without TRAIL and/or DMNB for the indicated times. The cells were centrifuged and resuspended in 500 μl of a staining solution containing Annexin V fluorescein (FITC Apoptosis Detection Kit; BD Pharmingen, San Diego, CA, USA) and propidium iodide in PBS. After incubation at room temperature for 15 min, the cells were analyzed by flow cytometry. Annexin V binds to cells that express phosphatidyl serine on the outer layer of their cell membrane, and propidium iodide stains the cellular DNA of cells with a compromised cell membrane. This allows for the discrimination of live cells (unstained with either fluorochrome) from apoptotic cells (stained only with Annexin V) and necrotic cells (stained with Annexin V and propidium iodide).

### Statistical analysis

The results obtained were expressed as the mean ± standard error (SE) from at least three independent experiments. The data were analyzed using the Student's t-test. *p <0.05, **p <0.01, and ***p <0.001 were considered statistically significant in all experiments.

## Abbreviations

TRAIL: tumor necrosis factor-related apoptosis-inducing ligand; DNA-PK: DNA-dependent protein kinase; siRNA: small interfering RNA; MTT: 3-(4, 5-dimethylthiazol-2-yl)-2,5-diphenyltetrazolium bromide; DMNB: 4,5-dimethoxy-2-nitrobenzaldehyde.

## Competing interests

The authors declare that they have no competing interests.

## Authors' contributions

HBK, MJK, DYK, JWL and JHB designed and conducted experiments as well data analysis. DWK participated in discussion of the data and draft of the manuscript. SHK and CDK equally participated in experimental design, coordination, data analysis and draft of the manuscript. All authors read and approved the final manuscript.

## References

[B1] Rosa DD Ismael G Lago LD Awada A Molecular-targeted therapies: lessons from years of clinical development Cancer Treat Rev 2008 34 61 80 10.1016/j.ctrv.2007.07.019 17826917

[B2] Cusack JC Jr Overcoming antiapoptotic responses to promote chemosensitivity in metastatic colorectal cancer to the liver Ann Surg Oncol 2003 10 852 862 10.1245/ASO.2003.07.518 14527902

[B3] Sorscher SM Biological therapy update in colorectal cancer Expert Opin Biol Ther 2007 7 509 519 10.1517/14712598.7.4.509 17373902

[B4] Mimeault M Batra SK Novel therapies against aggressive and recurrent epithelial cancers by molecular targeting tumor- and metastasis-initiating cells and their progenies Anticancer Agents Med Chem 2010 10 137 151 20184544 10.2174/187152010790909353 PMC2997522

[B5] Rudmik LR Magliocco AM Molecular mechanisms of hepatic metastasis in colorectal cancer J Surg Oncol 2005 92 347 359 10.1002/jso.20393 16299807

[B6] Merino D Lalaoui N Morizot A Solary E Micheau O TRAIL in cancer therapy: present and future challenges Expert Opin Ther Targets 2007 11 1299 1314 10.1517/14728222.11.10.1299 17907960 PMC2976473

[B7] Rowinsky EK Targeted induction of apoptosis in cancer management: the emerging role of tumor necrosis factor-related apoptosis-inducing ligand receptor activating agents J Clin Oncol 2005 23 9394 9407 10.1200/JCO.2005.02.2889 16361639

[B8] Kruyt FA TRAIL and cancer therapy Cancer Lett 2008 263 14 25 10.1016/j.canlet.2008.02.003 18329793

[B9] Fernandez T Torres MJ R RP Fuentes MS Robles S Mayorga C Blanca M Decrease of selective immunoglobulin E response to amoxicillin despite repeated administration of benzylpenicillin and penicillin V Clin Exp Allergy 2005 35 1645 1650 10.1111/j.1365-2222.2005.02378.x 16393332

[B10] Nieminen AI Partanen JI Klefstrom J c-Myc blazing a trail of death: coupling of the mitochondrial and death receptor apoptosis pathways by c-Myc Cell Cycle 2007 6 2464 2472 10.4161/cc.6.20.4917 17914284

[B11] Hoffman B Liebermann DA Apoptotic signaling by c-MYC Oncogene 2008 27 6462 6472 10.1038/onc.2008.312 18955973

[B12] Nieminen AI Partanen JI Hau A Klefstrom J c-Myc primed mitochondria determine cellular sensitivity to TRAIL-induced apoptosis EMBO J 2007 26 1055 1067 10.1038/sj.emboj.7601551 17268552 PMC1852827

[B13] Wang Y Engels IH Knee DA Nasoff M Deveraux QL Quon KC Synthetic lethal targeting of MYC by activation of the DR5 death receptor pathway Cancer Cell 2004 5 501 512 10.1016/S1535-6108(04)00113-8 15144957

[B14] Um JH Kwon JK Kang CD Kim MJ Ju DS Bae JH Kim DW Chung BS Kim SH Relationship between antiapoptotic molecules and metastatic potency and the involvement of DNA-dependent protein kinase in the chemosensitization of metastatic human cancer cells by epidermal growth factor receptor blockade J Pharmacol Exp Ther 2004 311 1062 1070 10.1124/jpet.104.070938 15273254

[B15] An J Yang DY Xu QZ Zhang SM Huo YY Shang ZF Wang Y Wu DC Zhou PK DNA-dependent protein kinase catalytic subunit modulates the stability of c-Myc oncoprotein Mol Cancer 2008 7 32 10.1186/1476-4598-7-32 18426604 PMC2383926

[B16] Qiao M Iglehart JD Pardee AB Metastatic potential of 21T human breast cancer cells depends on Akt/protein kinase B activation Cancer Res 2007 67 5293 5299 10.1158/0008-5472.CAN-07-0877 17545609

[B17] Kim SH Ricci MS El-Deiry WS Mcl-1: a gateway to TRAIL sensitization Cancer Res 2008 68 2062 2064 10.1158/0008-5472.CAN-07-6278 18381408

[B18] Ricci MS Kim SH Ogi K Plastaras JP Ling J Wang W Jin Z Liu YY Dicker DT Chiao PJ Reduction of TRAIL-induced Mcl-1 and cIAP2 by c-Myc or sorafenib sensitizes resistant human cancer cells to TRAIL-induced death Cancer Cell 2007 12 66 80 10.1016/j.ccr.2007.05.006 17613437

[B19] Ricci MS Jin Z Dews M Yu D Thomas-Tikhonenko A Dicker DT El-Deiry WS Direct repression of FLIP expression by c-myc is a major determinant of TRAIL sensitivity Mol Cell Biol 2004 24 8541 8555 10.1128/MCB.24.19.8541-8555.2004 15367674 PMC516765

[B20] Iijima S Teraoka H Date T Tsukada K DNA-activated protein kinase in Raji Burkitt's lymphoma cells. Phosphorylation of c-Myc oncoprotein Eur J Biochem 1992 206 595 603 10.1111/j.1432-1033.1992.tb16964.x 1597196

[B21] Lane D Robert V Grondin R Rancourt C Piche A Malignant ascites protect against TRAIL-induced apoptosis by activating the PI3K/Akt pathway in human ovarian carcinoma cells Int J Cancer 2007 121 1227 1237 10.1002/ijc.22840 17534891

[B22] Larribere L Khaled M Tartare-Deckert S Busca R Luciano F Bille K Valony G Eychene A Auberger P Ortonne JP PI3K mediates protection against TRAIL-induced apoptosis in primary human melanocytes Cell Death Differ 2004 11 1084 1091 10.1038/sj.cdd.4401475 15243584

[B23] Asakuma J Sumitomo M Asano T Hayakawa M Selective Akt inactivation and tumor necrosis actor-related apoptosis-inducing ligand sensitization of renal cancer cells by low concentrations of paclitaxel Cancer Res 2003 63 1365 1370 12649200

[B24] Xu J Zhou JY Wei WZ Wu GS Activation of the Akt survival pathway contributes to TRAIL resistance in cancer cells PLoS One 2010 5 e10226 10.1371/journal.pone.0010226 20419107 PMC2856686

[B25] Feng J Park J Cron P Hess D Hemmings BA Identification of a PKB/Akt hydrophobic motif Ser-473 kinase as DNA-dependent protein kinase J Biol Chem 2004 279 41189 41196 10.1074/jbc.M406731200 15262962

[B26] Teraoka H Yumoto Y Watanabe F Tsukada K Suwa A Enari M Nagata S CPP32/Yama/apopain cleaves the catalytic component of DNA-dependent protein kinase in the holoenzyme FEBS Lett 1996 393 1 6 10.1016/0014-5793(96)00842-3 8804412

[B27] Durant S Karran P Vanillins--a novel family of DNA-PK inhibitors Nucleic Acids Res 2003 31 5501 5512 10.1093/nar/gkg753 14500812 PMC206453

[B28] Seo SB Hur JG Kim MJ Lee JW Kim HB Bae JH Kim DW Kang CD Kim SH TRAIL sensitize MDR cells to MDR-related drugs by down-regulation of P-glycoprotein through inhibition of DNA-PKcs/Akt/GSK-3beta pathway and activation of caspases Mol Cancer 2010 9 199 10.1186/1476-4598-9-199 20663232 PMC2918570

[B29] Park SJ Bijangi-Vishehsaraei K Safa AR Selective TRAIL-triggered apoptosis due to overexpression of TRAIL death receptor 5 (DR5) in P-glycoprotein-bearing multidrug resistant CEM/VBL1000 human leukemia cells Int J Biochem Mol Biol 2010 1 90 100 20953314 PMC2953951

[B30] Hall MA Cleveland JL Clearing the TRAIL for Cancer Therapy Cancer Cell 2007 12 4 6 10.1016/j.ccr.2007.06.011 17613431

[B31] Pelengaris S Khan M Evan G c-MYC: more than just a matter of life and death Nat Rev Cancer 2002 2 764 776 10.1038/nrc904 12360279

[B32] Hueber AO Zornig M Lyon D Suda T Nagata S Evan GI Requirement for the CD95 receptor-ligand pathway in c-Myc-induced apoptosis Science 1997 278 1305 1309 10.1126/science.278.5341.1305 9360929

[B33] Klefstrom J Arighi E Littlewood T Jaattela M Saksela E Evan GI Alitalo K Induction of TNF-sensitive cellular phenotype by c-Myc involves p53 and impaired NF-kappaB activation EMBO J 1997 16 7382 7392 10.1093/emboj/16.24.7382 9405367 PMC1170338

[B34] Han J Goldstein LA Gastman BR Rabinowich H Interrelated roles for Mcl-1 and BIM in regulation of TRAIL-mediated mitochondrial apoptosis J Biol Chem 2006 281 10153 10163 10.1074/jbc.M510349200 16478725

[B35] Kataoka T Tschopp J N-terminal fragment of c-FLIP(L) processed by caspase 8 specifically interacts with TRAF2 and induces activation of the NF-kappaB signaling pathway Mol Cell Biol 2004 24 2627 2636 10.1128/MCB.24.7.2627-2636.2004 15024054 PMC371124

[B36] Shintani S Mihara M Li C Nakahara Y Hino S Nakashiro K Hamakawa H Up-regulation of DNA-dependent protein kinase correlates with radiation resistance in oral squamous cell carcinoma Cancer Sci 2003 94 894 900 10.1111/j.1349-7006.2003.tb01372.x 14556663 PMC11160163

[B37] Stronati L Gensabella G Lamberti C Barattini P Frasca D Tanzarella C Giacobini S Toscano MG Santacroce C Danesi DT Expression and DNA binding activity of the Ku heterodimer in bladder carcinoma Cancer 2001 92 2484 2492 10.1002/1097-0142(20011101)92:9<2484::AID-CNCR1598>3.0.CO;2-7 11745306

[B38] Tonotsuka N Hosoi Y Miyazaki S Miyata G Sugawara K Mori T Ouchi N Satomi S Matsumoto Y Nakagawa K Heterogeneous expression of DNA-dependent protein kinase in esophageal cancer and normal epithelium Int J Mol Med 2006 18 441 447 16865228

[B39] Moll U Lau R Sypes MA Gupta MM Anderson CW DNA-PK, the DNA-activated protein kinase, is differentially expressed in normal and malignant human tissues Oncogene 1999 18 3114 3126 10.1038/sj.onc.1202640 10340383

[B40] An J Xu QZ Sui JL Bai B Zhou PK Downregulation of c-myc protein by siRNA-mediated silencing of DNA-PKcs in HeLa cells Int J Cancer 2005 117 531 537 10.1002/ijc.21093 15929110

[B41] Puduvalli VK Sampath D Bruner JM Nangia J Xu R Kyritsis AP TRAIL-induced apoptosis in gliomas is enhanced by Akt-inhibition and is independent of JNK activation Apoptosis 2005 10 233 243 10.1007/s10495-005-6078-3 15711939 PMC3820101

[B42] Lee YJ Froelich CJ Fujita N Tsuruo T Kim JH Reconstitution of caspase-3 confers low glucose-enhanced tumor necrosis factor-related apoptosis-inducing ligand cytotoxicity and Akt cleavage Clin Cancer Res 2004 10 1894 1900 10.1158/1078-0432.CCR-03-0136 15041704

[B43] Affar EB Germain M Winstall E Vodenicharov M Shah RG Salvesen GS Poirier GG Caspase-3-mediated processing of poly(ADP-ribose) glycohydrolase during apoptosis J Biol Chem 2001 276 2935 2942 10.1074/jbc.M007269200 11053413

[B44] Graham KL Gustin KE Rivera C Kuyumcu-Martinez NM Choe SS Lloyd RE Sarnow P Utz PJ Proteolytic cleavage of the catalytic subunit of DNA-dependent protein kinase during poliovirus infection J Virol 2004 78 6313 6321 10.1128/JVI.78.12.6313-6321.2004 15163725 PMC416498

[B45] Kim SH Um JH Dong-Won B Kwon BH Kim DW Chung BS Kang CD Potentiation of chemosensitivity in multidrug-resistant human leukemia CEM cells by inhibition of DNA-dependent protein kinase using wortmannin Leuk Res 2000 24 917 925 10.1016/S0145-2126(00)00061-8 11086175

[B46] Ismail IH Martensson S Moshinsky D Rice A Tang C Howlett A McMahon G Hammarsten O SU11752 inhibits the DNA-dependent protein kinase and DNA double-strand break repair resulting in ionizing radiation sensitization Oncogene 2004 23 873 882 10.1038/sj.onc.1207303 14661061

[B47] Zhao Y Thomas HD Batey MA Cowell IG Richardson CJ Griffin RJ Calvert AH Newell DR Smith GC Curtin NJ Preclinical evaluation of a potent novel DNA-dependent protein kinase inhibitor NU7441 Cancer Res 2006 66 5354 5362 10.1158/0008-5472.CAN-05-4275 16707462

